# The Effect of Fluid Pre-loading on Vital Signs and Hemodynamic Parameters in a Porcine Model of Lipopolysaccharide-Induced Endotoxemia

**DOI:** 10.7759/cureus.43103

**Published:** 2023-08-07

**Authors:** Zachary R Bergman, Roy K Kiberenge, Richard Bianco, Gregory Beilman, Colleen M Brophy, Kyle M Hocking, Bret D Alvis, Eric S Wise

**Affiliations:** 1 Surgery, University of Minnesota School of Medicine, Minneapolis, USA; 2 Critical Care, Allina Health, Minneapolis, USA; 3 Surgery, Vanderbilt University Medical Center, Nashville, USA; 4 Surgery and Biomedical Engineering, Vanderbilt University Medical Center, Nashville, USA; 5 Anesthesiology and Biomedical Engineering, Vanderbilt University Medical Center, Nashville, USA

**Keywords:** hemodynamics, vasopressor, norepinephrine, vasoplegia, sepsis, endotoxemia

## Abstract

Background

Animal models of distributive hypotension and resuscitation allow the assessment of hemodynamic monitoring modalities and resuscitation strategies. The fluid-first paradigm for resuscitation is currently being challenged with clinical trials. In this investigation, venous return and perfusion are assessed, and full hemodynamics are characterized, in a porcine model of endotoxemic hypotension with and without fluid pre-loading.

Methods

Two groups of six pigs had the induction of standardized endotoxemic hypotension (“critical hypotension”). Group 1 underwent four 10 cc/kg crystalloid boluses, and Group 2 was not fluid pre-resuscitated. Both groups underwent progressive norepinephrine (NE) up-titration to 0.25 mcg/kg/minute over 30 minutes. Vital signs, central parameters, and laboratory values were obtained at baseline, “critical hypotension,” after each bolus and during NE administration.

Results

Endotoxemia decreased the systemic vascular resistance (SVR) in Group 1 (1031±106 dyn/s/cm^-5^ versus 738±258 dyn/s/cm^-5^; P=0.03) and Group 2 (1121±196 dyn/s/cm^-5^ versus 759±342 dyn/s/cm^-5^; P=0.003). In Group 1, the four fluid boluses decreased heart rate (HR), pulmonary capillary wedge pressure (PCWP), and central venous pressure (CVP) (P<0.05). No changes were observed in blood pressure, cardiac output (CO), or lactate. NE up-titration increased HR in Group 1 and decreased CVP in both groups. Higher final CVP (11 {3} versus 4 {4} mmHg; P=0.01) and PCWP (5 {1} versus 2 {2} mmHg; P=0.005) values were observed in Group 1 relative to Group 2, reflecting increased venous return.

Conclusions

Porcine endotoxemic hypotension and resuscitation were robustly characterized. In this model, fluid loading improved venous return with NE, though perfusion (CO) was preserved by increased NE-induced chronotropy.

## Introduction

Distributive shock is characterized by low systemic vascular resistance (SVR), with associated hypotension and malperfusion. To attempt to augment perfusion, this form of shock is considered hyperdynamic, as the heart is forced to increase its inotropic and chronotropic activities. Sepsis and septic shock due to bacterial infection are the most frequently encountered forms of distributive shock and the most common cause of mortality in the intensive care unit (ICU). Management principles have been driven by the Surviving Sepsis Guidelines, recently updated in 2021, in which treatment is tailored to the normalization of mean arterial pressure (MAP) (>65 mmHg) and the reduction of serum lactic acid levels [1]. Challenges in following these guidelines effectively remain. Excessive fluid resuscitation leads to pulmonary edema, abdominal compartment syndrome, organ failure, and increased mortality [2-4]. Insufficient volume leads to malperfusion syndromes, primarily acute kidney injury [5]. The fluid-first paradigm to optimize perfusion has however persisted through multiple updates to the Surviving Sepsis Guidelines, as high-level evidence supports the institution of vasoconstricting medications, norepinephrine (NE) in particular, as a second-line “rescue” therapy.

While distinct from bacterial sepsis, endotoxemia due to bloodstream seeding with lipopolysaccharides (LPS) similarly produces low-SVR hypotension [6,7]. Models of endotoxemic shock have successfully been used to mimic the hemodynamic perturbations seen in septic shock [8]. LPS-associated hypotension in pigs is recognized, among other surgical models of sepsis, as a suitable, validated mammalian model of distributive shock with hemodynamic changes sufficiently similar to those seen in human sepsis [9,10]. A drawback of this model includes an initial low-dose “sensitization” response characterized by cytokine release, causing transient hemodynamic instability potentially requiring an epinephrine bolus. Strengths, which far outweigh the drawback, include the quick onset of hemodynamic changes, the ability to control the degree of shock by dose adjustment (“on/off”), reproducibility, clinical applicability, and low cost.

Large animal models of endotoxemia are highly useful to clinicians and scientists alike, as they can be used to assess resuscitation strategies, test novel hemodynamic monitoring modalities, and characterize perturbations seen in distributive hypotension and shock [10,11]. The robust characterization of these models further necessitates the placement of a pulmonary artery catheter (PAC), which is invasive and cumbersome and incurs risks of complications. PAC-derived parameters reflect “gold standard” measures of hemodynamics. The importance of cardiac output (CO), among the best clinical measures of perfusion, is underscored by the development of multiple less-invasive devices such as LiDCO or PiCCO to estimate it, though these devices also require restrictive conditions such as paralysis and high tidal volume ventilation [12]. Additionally, pulmonary capillary wedge pressure (PCWP) is a critical measure adequacy of venous return to the right heart, and the plausibility of its use as a titratable endpoint has prompted the development of non-invasive surrogates [13,14]. Pulmonary arterial pressures, which can become elevated in sepsis or endotoxemia, can also be measured. In this investigation, using a previously validated model of endotoxemic hypotension, a full characterization of hemodynamic parameters is reported [8]. Uniquely, vital signs, central hemodynamic parameters, and key laboratory measures are compared after controlled resuscitation with NE, both with and without controlled crystalloid pre-resuscitation.

## Materials and methods

To induce low-SVR hypotension, a previously reported porcine model of intravenous (IV) LPS infusion was used [8]. In this model, low doses of LPS are known to be associated with an initial increase in pulmonary arterial pressures. After initial instability and pausing of the LPS, pulmonary arterial pressures normalize, and more stable distributive hypotension at higher LPS doses is precipitated. An up-titration protocol of LPS was used to cause the desired respiratory insult. As noted previously, this study used 12 female (to avoid potentially exaggerated LPS response notes in male pigs) Yorkshire/Landrace pigs (~12 weeks of age; Manthei Hog Farm, LLC, Elk River, MN), under a protocol approved by the University of Minnesota Institutional Animal Care and Use Committee, divided into two groups. All Animal Research: Reporting of In Vivo Experiments (ARRIVE) guidelines and those set forth by the National Institutes of Health (NIH) for the care and use of laboratory animals were followed. The sample size was determined considering the principle of reduction and the sample size used in prior analogous protocols, without the need for blinding or randomization, and six pigs were assigned to immediate NE treatment, while six were assigned to a moderate fluid pre-loading protocol.

Induction and preparation

The protocol of LPS infusion represents a slight adaptation of the method reported by Byrne and colleagues [15] and has been reported previously [8]. These experiments were performed under direct veterinary supervision in the designated animal operating room at the University of Minnesota Experimental Surgical Services. The pigs were initially sedated with intramuscular injection of ketamine (2.2 mg/kg)/xylazine (2.2 mg/kg)/Telazol (6 mg/kg) and intravenously catheterized. IV propofol was administered to effect if needed to allow intubation. Maintenance anesthesia was used with 3% isoflurane until the pigs were fully catheterized and then reduced to 2% isoflurane. Ventilatory settings were held at volume control mode with a tidal volume of 10-15 mL/kg, 10-15 breaths/minute, and positive end expiratory pressure at 4 mmHg, on 100% oxygen [8]. Anesthesia was maintained with 1%-4% inhaled isoflurane. The placement of the carotid arterial line and internal jugular pulmonary artery catheters via neck cut-down was as previously described [16]. All catheters were used to continuously transduce the arterial, central venous, and pulmonary arterial tracings, using LabChart 8 (ADInstruments, Sydney, Australia) [16].

LPS infusion

Once fully anesthetized and catheterized, pigs were brought to a euvolemic state using IV PlasmaLyte A (Baxter, Deerfield, IL) infusion to a pulmonary capillary wedge pressure (PCWP) of 10±1 mmHg, followed by a 15-minute equilibration period. Baseline vital signs (heart rate {HR}, systolic blood pressure {SBP}, diastolic blood pressure {DBP}, and MAP), arterial line-derived arterial blood gasses (to obtain arterial pH and lactate), PAC-obtained venous blood gasses (to obtain central venous oxygenation {ScvO_2_}; blood obtained from the superior vena cava), and central hemodynamic parameters (CO, PCWP, systolic pulmonary artery pressure {SPAP}, diastolic pulmonary artery pressure {DPAP}, mean pulmonary artery pressure {MPAP}, and central venous pressure {CVP}) were obtained. A lipopolysaccharide (Escherichia coli O111:B4, Sigma, St. Louis, MO) solution was initiated using the dosing protocol reported in Table 1, while all waveforms were being transduced.

**Table 1:** Dose titration protocol of lipopolysaccharides (LPS) during porcine experiments

Rate of LPS Infusion	Incremental Increase		Time Between Dose Increase
0.25 µg/kg/hour	Double rate	Every five minutes	
2 µg/kg/hour	1 µg/kg/hour	Every five minutes	
10 µg/kg/hour	2 µg/kg/hour	Every five minutes	
20 µg/kg/hour	5 µg/kg/hour	Every three minutes	
60 µg/kg/hour	10 µg/kg/hour	Every three minutes	

Significant hemodynamic instability is precipitated reliably between 5 and 7 µg/kg/hour LPS [8]. The point at which the MPAP was within 10 mmHg of MAP was taken as the point of imminent significant hemodynamic compromise, and LPS was paused while the pulmonary pressures normalized. At this point, the pigs were allowed to recover with resuscitation; seven pigs (n=3 in Group 1; n=4 in Group 2) required a dose of epinephrine for stabilization (5-20 µg). After ensuring the normalization of hemodynamics, LPS was then resumed until a more stable “critical hypotension,” defined as a 25% decrement from baseline MAP, was reached [8].

Fluid and vasoconstrictor interventions

**Figure 1:**
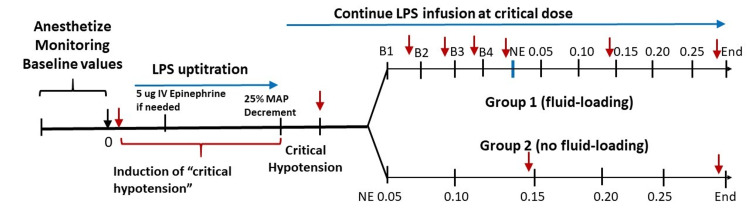
Diagrammatic representation of the porcine experiments (Group 1 and Group 2) Red arrows indicate points at which hemodynamic measurements were obtained. Units of norepinephrine (NE) dosing: mcg/kg/minute LPS, lipopolysaccharide; IV, intravenous; MAP, mean arterial pressure

The experimental protocol is outlined in Figure 1. In the fluid-first group, four boluses of 10 cc/kg PlasmaLyte A (chosen for increased granularity of the data) were incrementally administered to the pigs, prior to NE institution. Full vital signs and central hemodynamic parameters were obtained after each bolus; blood samples were obtained after the first two and final two boluses, prior to NE administration. In the NE-first group, the fluid loading was omitted. Both groups underwent an NE infusion protocol, with LPS at the “critical” dose still infusing as well. NE was initiated at 0.05 mcg/kg/minute. NE was subsequently increased every five minutes until a dose of 0.25 mcg/kg/minute was reached, and this dose was allowed to infuse for five additional minutes. Vital signs and PAC-derived hemodynamic parameters were obtained just before NE institution, just before 0.15 mcg/kg/minute, and at five minutes of the dose 0.25 mcg/kg/minute. Additionally, arterial and venous blood gasses were obtained before and after NE administration [8].

After the protocol was complete, pigs were humanely euthanized with IV sodium pentobarbital (125 mg/kg).

Data analysis

Measures of central tendency are reported as mean (standard deviation), and comparisons between the two groups were made using paired or unpaired Student’s t-tests, as appropriate. One-way analysis of variance (ANOVA) with post hoc tests was used to assess parameter changes over the course of fluid or pressor administration. A criterion of P≤0.05 was used to denote statistical significance.

Most hemodynamic values were directly measured. MPAP was taken as (2/3)(DPAP)+(1/3)(SPAP). SVR was calculated using 80*(MAP-CVP)/CO. CO was taken as the average of three thermodilution-derived measurements. PCWP was obtained at the end of expiration. Arterial waveforms, central venous pressure waveforms, and pulmonary artery waveforms were continually transduced to LabChart 8 for post hoc analysis.

## Results

The precipitation of critical hypotension was achieved without mortalities in all 12 pigs, in the two groups. Mean SVRs for the two groups (fluid loading, Group 1; no fluid loading, Group 2) significantly decreased from 1031 (106) dyn/s/cm^-5^ to 738 (258) dyn/s/cm^-5^ and 1121 (196) dyn/s/cm^-5^ to 759 (342) dyn/s/cm^-5^, respectively, indicating low-resistance hypotension. Vital signs and central hemodynamic parameters are summarized in Table 2. Both groups demonstrated significant increases in HR, SPAP, DPAP, MPAP, and lactate at critical hypotension, relative to baseline. Both groups also demonstrated significant decreases in SBP, DBP, MAP, and arterial pH. No changes were detected in CVP, CO, and ScvO_2_; Group 1 did demonstrate a significant decrease in PCWP (10 {0.6} mmHg versus 8 {1} mmHg; P=0.01), whereas Group 2 did not (10 {0} mmHg versus 6 {4} mmHg; P=0.09). Baseline parameters were well matched, though Group 1 had a slightly decreased DPAP and MPAP relative to Group 2.

**Table 2:** Vital signs and central hemodynamic parameters at baseline and after the precipitation of critical hypotension (CH) in the two groups Significant differences in analogous measurements (both at baseline and at “critical hypotension”) between Group 1 and Group 2 cohorts were assessed via unpaired t-test (*P<0.05) SVR, systemic vascular resistance; ScvO_2_, central venous oxygenation

	Group 1 (n=6), Fluid Loading			Group 2 (n=6), No Fluid Loading		
Vital sign	Baseline	CH	P (paired t)	Baseline	CH	P (paired t)
Heart rate (beats per minute)	86 (14)	110 (9)	0.006	92 (12)	118 (14)	0.01
Systolic blood pressure (mmHg)	89 (15)	65 (8)	<0.001	91 (18)	70 (11)	0.01
Diastolic blood pressure (mmHg)	56 (11)	29 (6)	<0.001	59 (16)	31 (9)	<0.001
Mean arterial pressure (mmHg)	71 (13)	43 (6)	<0.001	73 (18)	45 (7)	0.002
Central hemodynamic parameters						
Systolic pulmonary arterial pressure (mmHg)	23 (2)	35 (6)	0.002	24 (4)	35 (4)	0.007
Diastolic pulmonary arterial pressure (mmHg)	10 (2)*	19 (6)	0.01	14 (3)	24 (7)	0.03
Mean pulmonary arterial pressure (mmHg)	13 (1)*	24 (4)	0.001	15 (2)	28 (6)	0.006
Pulmonary capillary wedge pressure (mmHg)	10 (0.6)	8 (1)	0.01	10 (0)	6 (4)	0.09
Central venous pressure (mmHg)	5 (2)	5 (2)	0.62	5 (0.8)	4 (2)	0.66
Cardiac output (L/minute)	5.2 (1.2)	4.5 (1.3)	0.33	5.0 (1.4)	5.0 (1.9)	0.98
SVR	1031 (106)	738 (258)	0.03	1121 (196)	759 (342)	0.003
Additional values						
Lactate (mmol/L)	2.0 (0.4)	4.0 (1.3)	0.006	1.8 (0.7)	3.7 (1.6)	0.01
Arterial pH	7.48 (0.07)	7.40 (0.04)	0.005	7.51 (0.04)	7.41 (0.03)	0.001
ScvO_2_	84 (4)	84 (6)	1.00	81 (9)	82 (5)	0.63

During the course of four 10 cc/kg boluses, HR decreased (P=0.02), though no changes in systemic or pulmonary blood pressures were noted. PCWP (P=0.0003) and CVP (<0.001) were central parameters that increased in response to fluid. Over the range of fluid delivered, CO elevation was observed relative to critical hypotension only upon the post hoc analysis of multiple comparisons, after bolus 2. There was evidence of a trend indicative of increasing CO during the course of the fluid loading (P=0.09). Hemodynamic parameters and select laboratory values obtained during the fluid-loading protocol in Group 1 are summarized in Table 3.

**Table 3:** Vital signs and central hemodynamic parameters at baseline and over the course of fluid resuscitation from critical hypotension (CH), Group 1 (during fluid pre-loading) Significant differences upon one-way analysis of variance (ANOVA) with post hoc comparison to CH are acknowledged (*P<0.05; †P<0.01; ‡P<0.001) SVR, systemic vascular resistance; ScvO_2_, central venous oxygenation

	Group 1 (n=6), Fluid Loading					
Vital sign	CH	Post-bolus 1	Post-bolus 2	Post-bolus 3	Post-bolus 4	P
Heart rate (beats per minute)	110 (9)	103 (13)	101 (13)	97 (10)*	99 (13)	0.02
Systolic blood pressure (mmHg)	65 (8)	61 (8)	67 (12)	63 (13)	69 (10)	0.53
Diastolic blood pressure (mmHg)	29 (6)	28 (7)	27 (7)	27 (6)	31 (6)	0.60
Mean arterial pressure (mmHg)	43 (6)	43 (10)	45 (10)	47 (8)	48 (7)	0.41
Central hemodynamic parameters						
Systolic pulmonary arterial pressure (mmHg)	35 (6)	40 (7)	42 (7)	49 (19)	45 (6)^†^	0.18
Diastolic pulmonary arterial pressure (mmHg)	19 (6)	23 (3)	22 (6)^†^	23 (9)	22 (4)	0.56
Mean pulmonary arterial pressure (mmHg)	24 (4)	29 (4)^‡^	29 (6)^†^	32 (12)	30 (3)	0.32
Pulmonary capillary wedge pressure (mmHg)	8 (1)	9 (0.8)	11 (1)^†^	14 (4)*	14 (3)*	0.003
Central venous pressure (mmHg)	5 (2)	7 (3)	10 (3)^†^	11 (4)^†^	11 (2)^†^	<0.001
Cardiac output (L/minute)	4.5 (1.3)	5.2 (1.1)	5.3 (1.5)*	5.8 (1.3)	5.4 (1.3)	0.09
SVR	738 (258)	551 (160)	575 (252)	494 (104)	556 (166)	0.04
Additional values						
Lactate (mmol/L)	4.0 (1.3)		3.8 (1.0)		4.1 (0.8)	0.40
Arterial pH	7.40 (0.04)		7.36 (0.04)^‡^		7.35 (0.05)^†^	<0.001
ScvO_2_ (n=5)	84 (6)		83 (12)		79 (7)	0.24

During the NE infusion protocol, HR significantly increased in Group 1, the fluid-loaded pigs (P=0.003), though was not changed in the NE-only pigs. The final HRs for both groups were not different. Notably, in this model, MAP was not augmented with NE infusion. In both groups, NE administration generated a decrease in CVP, though without significant changes in other central parameters. At the end of the resuscitation protocol, both PCWP (11 {3} mmHg versus 4 {4} mmHg; P=0.01) and CVP (5 {1} mmHg versus 2 {2} mmHg; P=0.005) were higher in Group 1 than in Group 2. These results are summarized in Table 4.

**Table 4:** Vital signs and central hemodynamic parameters over the course of norepinephrine (NE) resuscitation in the two groups Group 1 was fluid pre-loaded. Significantly different from baseline on post hoc test, compared to baseline (*P<0.05; †P<0.01; ‡P<0.001). Units of norepinephrine (NE) dosing: mcg/kg/minute SVR, systemic vascular resistance; ScvO_2_, central venous oxygenation

	Group 1 (n=6), Fluid Loading				Group 2 (n=6), No Fluid Loading				Comparison of End Values
Vital sign	Pre-NE	Pre-NE 0.15	End	P	Pre-NE	Pre-NE 0.15	End	P	P (unpaired t-test)
Heart rate (beats per minute)	99 (13)	115 (14)*	132 (16)^†^	0.003	118 (14)	124 (7)	140 (14)	0.07	0.36
Systolic blood pressure (mmHg)	69 (10)	76 (15)	80 (2)	0.22	70 (11)	72 (11)	84 (16)	0.12	0.71
Diastolic blood pressure (mmHg)	31 (6)	32 (9)	31 (10)	0.81	31 (9)	32 (15)	33 (21)	0.73	0.86
Mean arterial pressure (mmHg)	48 (7)	49 (10)	49 (14)	0.12	45 (7)	47 (12)	53 (20)	0.35	0.68
Central hemodynamic parameters									
Systolic pulmonary arterial pressure (mmHg)	45 (6)	42 (6)	38 (8)	0.13	35 (4)	38 (9)	38 (7)	0.45	1.0
Diastolic pulmonary arterial pressure (mmHg)	22 (4)	21 (8)	22 (3)	0.90	24 (7)	25 (10)	25 (6)	0.79	0.80
Mean pulmonary arterial pressure (mmHg)	30 (3)	28 (6)	27 (4)	0.51	28 (6)	29 (9)	29 (6)	0.63	0.78
Pulmonary capillary wedge pressure (mmHg)	14 (3)	9 (4)	11 (3)	0.052	6 (4)	5 (4)	4 (4)	0.14	0.01
Central venous pressure (mmHg)	11 (2)	5 (1)^‡^	5 (1)^‡^	<0.001	4 (2)	3 (3)	2 (2)	0.03	0.005
Cardiac output (L/minute)	5.4 (1.3)	6.1 (1.9)	5.8 (2.3)	0.61	5.0 (1.9)	5.0 (1.3)	5.5 (0.9)	0.37	0.81
SVR	556 (166)	602 (128)	639 (141)	0.40	759 (342)	778 (393)	754 (313)	0.72	0.43
Additional values									
Lactate (mmol/L)	4.1 (0.8)		4.7 (0.9)	0.14	3.7 (1.6)		4.1 (1.2)	0.31	0.36
Arterial pH	7.35 (0.05)		7.35 (0.08)	0.85	7.41 (0.03)		7.37 (0.03)	0.007	0.58
ScvO_2_	79 (7)		83 (12)	0.42	79 (8)		84 (5)	0.27	0.90

## Discussion

In this investigation, a thorough characterization of a porcine model of endotoxemic hypotension and shock is provided. Moreover, changes in vital signs, central hemodynamic parameters, and key laboratory values indicative of the severity of hypotension and shock are reported during the course of initial fluid resuscitation and during NE-based resuscitation. Clinical paradigms warrant intravenous crystalloid fluid as the initial treatment to increase blood pressure, restore intravascular volume, optimize venous return, and increase perfusion. However, the parameters assessed in this model have not been well characterized in the case where distributive hypotension is treated first with NE, the first-line clinically employed vasopressor. The principal findings are not only that this model of endotoxemia can reliably propagate low-resistance, distributive hypotension but also that fluid pre-loading ultimately increases key parameters indicative of a venous return to the right heart: CVP and PCWP.

Multiple other reports of porcine endotoxemia use a constant dose of LPS administration. Endo and colleagues infused LPS at a constant rate of 80 µg/kg/hour. While direct PAC data were not available, interestingly, changes were not observed in HR, MAP, or CVP relative to their baseline values, or a control cohort, during the course of the experiment, while SVR (using PiCCO to estimate CO) only declined after 60 minutes, with normalization by 90 minutes [7]. As these authors point out, a dynamic change in LPS infusion rate is necessary to model sepsis in these pigs, as was intentionally done in this experiment [17]. Caution must be taken to avoid pulmonary edema, which typically manifests at sustained higher LPS doses; pulmonary edema was not noted on lung necropsy on any of the pigs [18]. Additionally, the timing of resuscitation of endotoxemic shock was assessed by Gomez and colleagues, who did sequentially up-titrate LPS. Despite finding pigs not surviving the “LPS priming” period, they found, similar to the findings herein, a steady trend of decline in MAP and CO during LPS infusion; HR, CVP, MPAP, and PCWP parameters were not reported. Their findings showed that protocolized resuscitation using a fluid-first paradigm blunted these deleterious hemodynamic perturbations when instituted early [19]. While, ideally, resuscitation would begin upon the onset of distributive hypotension to avoid malperfusion, the data from this investigation must be interpreted in the common clinical scenario of the recognition of hypotension after an insult, with the institution of fluid (or NE-based) resuscitation in short order.

This study underscores the sensitivity of PCWP and CVP during fluid- or pressor-based resuscitation from states of distributive shock. PCWP is recognized as a “gold standard” direct measure of fluid status. It is derived via the occlusion of the pulmonary artery with a balloon-tipped port of a PAC and provides an accurate measure of left atrial and left ventricular end-diastolic pressures [20]. Its use has been mitigated in cases such as hemorrhagic shock or sepsis, due to the necessity of a PAC, which is cumbersome. However, it is valuable in assessing fluid status after cardiac surgery, pulmonary hypertension, and heart failure, via right heart catheterization [21]. In hemorrhage and fluid resuscitation, PCWP, more than any parameter, exhibits the strongest linear correlation to the degree of fluid removed or replaced [17]. PCWP is imperfect, however, as its usefulness is decreased in cases of diastolic dysfunction, chronic obstructive pulmonary disease, positive pressure ventilation, obesity, and severe valvulopathy [22]. Nonetheless, the sensitivity of PCWP throughout moderate fluid administration and vasopressor up-titration provides further impetus to develop clinically facile less-invasive “PCWP” equivalent measurements used to detect and monitor right heart filling during critical illness and resuscitation [13,14,23]. This objective has been studied primarily by using peripherally obtained arterial or venous waveform analytic methods to deduce central parameters (e.g., compensatory reserve index and non-invasive venous waveform analysis). CVP, another indicator of venous return, which was found to change significantly through the course of fluid and pressor resuscitation, is easier to obtain, as it is transducible through an upper extremity central venous catheter. However, the use of CVP is no longer advocated to estimate volume responsiveness and volume status in sepsis resuscitation due to its (1) inaccuracy, (2) lack of improvement in sepsis outcomes, and (3) inherent zeroing error [24].

The importance of CO as a surrogate for perfusion, an important endpoint in resuscitation from distributive hypotension and shock, has led to the development of multiple devices to obtain a CO estimation without a PAC. These approaches for the estimation of CO either are operator-dependent (echocardiography) or require an arterial line, intubation, and high tidal volume ventilation for accuracy (pulse pressure variation, surrogates of thermodilution CO {e.g., “FloTrac,” “LiDCO,” and “PiCCO” monitors}) [12]. In this study, CO did not change after the NE infusion protocol between the two groups. Without fluid pre-loading, other mechanisms were ostensibly invoked to preserve CO [8]. CO augmentation by NE in distributive shock is thought to work by several mechanisms. Heart rate increase, a primary mechanism of NE, can increase CO if the HR is not prohibitively high such that filling time is decreased [25]. Additionally, cardiac inotropy is increased due to improved coronary perfusion, as well as the Anrep response, in which intrinsic contractility is improved [26]. Finally, improved NE-induced arterial elastance can improve ventriculoatrial coupling, thus increasing CO [25]. While fluid-first approaches in distributive hypotension are justified with high-level evidence, the change in CO induced by NE in fluid-responsive patients is a useful information, particularly when treating those patients who are highly fluid-sensitive, such as those with end-stage renal disease or severe baseline pulmonary compromise. The pattern of changes noted in vital signs and central hemodynamic parameters during the course of progressive distributive hypotension and shock is similar to other types of shock such as hemorrhage. Over the course of controlled porcine hemorrhage of 500 mL, PCWP and CVP decreased significantly, while CO was preserved via compensatory mechanisms [16].

The degree to which NE-induced CO augmentation is dependent on fluid pre-loading is assessed in a series of pigs to explore whether an up-front NE approach to increasing CO is plausible, a challenge to the current paradigm yet also a question that has prompted national studies. Early NE administration is thought to confer benefits including the prevention of prolonged hypotension, early CO increase, improved microcirculation (which most proximally governs vital organ perfusion), and the prevention of fluid overload [27]. Thus, fluid-dominant paradigms have recently been challenged in pivotal clinical trials, to elucidate the possible role of early pressor use in sepsis. Reported in 2018, results from the REFRESH randomized controlled trial revealed that limiting crystalloid infusion to 1 L prior to NE institution led to a reduction in overall fluid infused, without the evidence of clinical harm [28]. Based in Scotland, the “Early Vasopressors in Sepsis” (EVIS) trial is an ongoing phase 3 randomized prospective study implementing early peripheral NE infusion for septic hypotension, with adjunctive fluid only as needed, compared to standard care. The primary endpoint is all-cause 30-day mortality, with key secondary endpoints including lactate clearance and validated organ dysfunction scores. Most notably, the results of the NIH-funded CLOVERS study were recently reported in the New England Journal of Medicine [29]. In this randomized controlled trial, patients with septic hypotension refractory to initial fluid loading were randomized to liberal IV fluid or restrictive fluid (vasopressor use), with no change in mortality, or discharged by day 90 [29].

This model of swine endotoxemia may be useful for researchers and clinicians, as it allows for the mimicry of the physiology of sepsis and can be used accordingly. It is replicable and relatively cost-effective and allows for the assessment of alternative resuscitation strategies such as a “vasopressor-first” approach. The findings suggest, similar to hemorrhage, that compensatory mechanisms are effectively invoked to preserve CO during the controlled LPS insult, even in cases where pigs are significantly volume-responsive, yet undergo no fluid pre-loading. These data, coupled with the benefits of fluid-restrictive strategies, suggest the need for custom-tailoring resuscitation from distributive shock, as well as the mandate for non-invasive, accurate adjuncts to surrogate for the central parameters PCWP and CO.

Limitations

This model of endotoxemia causes a well-described, initial rapid elevation in pulmonary pressures leading to potential hemodynamic compromise requiring epinephrine, a well-recognized phenomenon whose mitigation with sildenafil has been attempted. Though this elevation in pulmonary pressures is transient and fully reversible, this critical event makes the findings less translatable to the more common human condition of septic shock [8]. Additionally, sample sizes of six per group may represent restricted cohorts that did not fully demonstrate parameter differences, due to heterogeneity in response to LPS infusion. To this effect, the use of healthy pigs of one gender, while justifiable and necessary for replicability, may lead to the lack of generalizability of the observed hemodynamic profiles. Patients with sepsis in the ICU are also variably intubated, sedated, and paralyzed, and thus, parameters in swine with only moderate sedation, or those intubated but not paralyzed, are warranted. Finally, these data are purely descriptive in nature; as a non-survival protocol, no information on the severity of organ malperfusion or post-LPS survival (outcomes) was obtained between the fluid-first and vasopressor group cohorts. Despite these limitations, these data represent a novel large animal characterization of porcine endotoxemia, as well as an interesting study of the consequences of the omission of fluid pre-loading on vital signs and hemodynamic parameters.

## Conclusions

This porcine model of LPS-induced endotoxemic hypotension with resuscitation can mimic human physiologic perturbations in sepsis. Using this model, a resuscitative approach with NE only generated a lower overall CVP and PCWP, while CO was similar after fluid pre-loading. Further animal and human studies are warranted to optimize resuscitation from distributive shock, balancing excess fluid with the risk of organ malperfusion.
